# Social Network Sites as a Mode to Collect Health Data: A Systematic Review

**DOI:** 10.2196/jmir.3050

**Published:** 2014-07-14

**Authors:** Fahdah Alshaikh, Farzan Ramzan, Salman Rawaf, Azeem Majeed

**Affiliations:** ^1^School of Public HealthDepartment of Primary Care and Public HealthImperial College LondonLondonUnited Kingdom

**Keywords:** social network, social media, Internet, systematic review, young people, health education, health behaviors

## Abstract

**Background:**

To date, health research literature has focused on social network sites (SNS) either as tools to deliver health care, to study the effect of these networks on behavior, or to analyze Web health content. Less is known about the effectiveness of these sites as a method for collecting data for health research and the means to use such powerful tools in health research.

**Objective:**

The objective of this study was to systematically review the available literature and explore the use of SNS as a mode of collecting data for health research. The review aims to answer four questions: Does health research employ SNS as method for collecting data? Is data quality affected by the mode of data collection? What types of participants were reached by SNS? What are the strengths and limitations of SNS?

**Methods:**

The literature was reviewed systematically in March 2013 by searching the databases MEDLINE, Embase, and PsycINFO, using the Ovid and PubMed interface from 1996 to the third week of March 2013. The search results were examined by 2 reviewers, and exclusion, inclusion, and quality assessment were carried out based on a pre-set protocol.

**Results:**

The inclusion criteria were met by 10 studies and results were analyzed descriptively to answer the review questions. There were four main results. (1) SNS have been used as a data collection tool by health researchers; all but 1 of the included studies were cross-sectional and quantitative. (2) Data quality indicators that were reported include response rate, cost, timeliness, missing data/completion rate, and validity. However, comparison was carried out only for response rate and cost as it was unclear how other reported indicators were measured. (3) The most targeted population were females and younger people. (4) All studies stated that SNS is an effective recruitment method but that it may introduce a sampling bias.

**Conclusions:**

SNS has a role in health research, but we need to ascertain how to use it effectively without affecting the quality of research. The field of SNS is growing rapidly, and it is necessary to take advantage of the strengths of this tool and to avoid its limitations by effective research design. This review provides an important insight for scholars who plan to conduct research using SNS.

##  Introduction

### Overview

Since their introduction, social network sites (SNS) have attracted individuals, businesses, social organizations, and lately health organizations and providers. There are millions of users, each with a different purpose for using these networks.

The purpose of this review is to focus on those networks that are defined as “Web-based services that allow individuals to construct a public or semi-public profile within a bounded system, articulate a list of other users with whom they share a connection, and view and traverse their list of connections and those made by others within the system” [1].

Social network sites and social media include all types of online social platforms that allow participants to share interests and opinions and many other social interactions. The use of these platforms is becoming dominant among all Internet usage purposes, and today Web content often has the feature to share or link to SNS. It seems that the importance of a topic is linked to its presence in SNS [2].

Social networking is not just about being on a website. It comprises a community that shares and interacts. It is a powerful community that has shifted the concept of media and is rapidly and extensively penetrating society [3].

### Social Network Sites in Health Research

For researchers, SNS is an environment where sharing information, knowledge, interest, and opinion is meaningful and fun, which makes it ideal for conducting research [2]. The promise that SNS held for health has been explored and discussed in previous publications. SNS may play a role for health in two ways: (1) the presence of health organizations on SNS makes them more approachable and accessible, and (2) SNS may be an effective way of helping patients with chronic diseases manage their health conditions. The importance of SNS is reflected by increasing efforts within health sectors and organizations to embrace SNS [3,4].

However, efforts towards using SNS are still in their infancy and more inventive interventions and other ways of benefiting from SNS are yet to be explored and discussed [3]. One of the many possible uses of SNS for health is using this powerful platform to collect data and recruit for research studies.

### Potential of Social Network Sites for Data Collection

Interest in online social networks has been increasing over the past few years as a result of the huge adoption of this technology all over the world.

The literature shows that SNS has been used by researchers as a source of information about user characteristics, patterns of friending, and usage behavior [5]. These social networks have become a modern source for information and data gathering. They have evolved into a dynamic and accurate source of gathering information because they contain a feature not found in traditional media: active and two-way participation [6].

SNS are also extraordinary marketing tools, able to reach almost any type of person, which changes communication from “one-to-one” to “many-to-many” [7]. Finally, they have become sources of collecting timely information, converting data into profitable results at a faster rate. They contain great opportunities for future research in public health because they can be a great way to reach hidden and hard-to-reach groups [8]. Yet there is still relatively little direction on how SNS can be used in health research and whether they can provide valid and reliable data.

### Research Objectives

The aim of this study was to systematically review the available literature and explore the use of SNS as a mode of collecting data for health research. The review aims to answer four questions: (1) Does health research employ SNS as a mode of collecting data? (2) Is data quality affected by the mode of data collection? (3) What types of participants were reached by SNS? (4) What are the strengths and limitations of SNS?

## Methods

### Systematic Review

The literature was reviewed systematically by searching bibliographic databases, MEDLINE, Embase, and PsycINFO, in March 2013 using the Ovid and PubMed interface for the period 1996 to the third week of March 2013, using the following keyword combinations: (Online Social Networks or Online Social Sites or Social Media) AND (Health). In addition, a manual search was undertaken, searching the reference list of all included studies.

The review was conducted by 2 reviewers independently. Search results were extracted to an Endnote database, inclusion and exclusion processes were recorded, and all abstracts and titles were reviewed. The initial selection criteria were (1) Intervention: The review focuses specifically on using SNS as a mode of collecting data, rather than as a social intervention (eg, support group), (2) Time and place: Studies produced at any time and place will be included in the search strategy, (3) Study participants: can include community or patients, and (4) Outcomes: included studies must contain outcomes related to the data collection mode, for example, response rate, completeness, missing data, timeliness, cost, and perception of privacy and anonymity. There were no language restrictions to ensure that as many studies as possible were assessed for relevance to the review.

Studies were excluded if they examined SNS participant interaction rather than SNS as a mode of collecting data, if they did not involve SNS, or if the article was a general discussion paper that did not present data or methods. All included studies had to specify the use of SNS as a tool to collect self-reported health data.

The review focused on the quality of data, the strengths and limitations of the mode, and reported strategies that facilitate the data collection. After inclusion based on title and abstract, full articles were retrieved and data extracted with a predesigned extraction form that included a checklist to assess the quality of each included study. The checklist was developed by the Centre for Reviews and Dissemination (CRD) [9]. Studies that scored 5 out of 7 or below were considered as low quality, and above 5 were considered as high quality.

### Data Analysis

The wide variety of methodologies and outcomes of included studies limited the possibility to carry out meta-analyses. For example, study populations were different and for the studies with similar populations, they had different methods. A descriptive qualitative analysis was carried out to answer the four research questions.

The PRISMA (Preferred Reporting Items for Systematic reviews and Meta-Analyses) checklist was followed in this systematic review [10]. The checklist items are essential for transparent reporting of a systematic review, and the author covered most of these items except items related to meta-analysis, which was not applicable for this review (Multimedia Appendix 1).

## Results

### Overview

A total of 1534 citations were identified using the search strategy from the electronic databases and search results combined with articles identified by searching manually with duplicates removed; 1213 citation titles and abstracts were reviewed.

**Figure 1:**
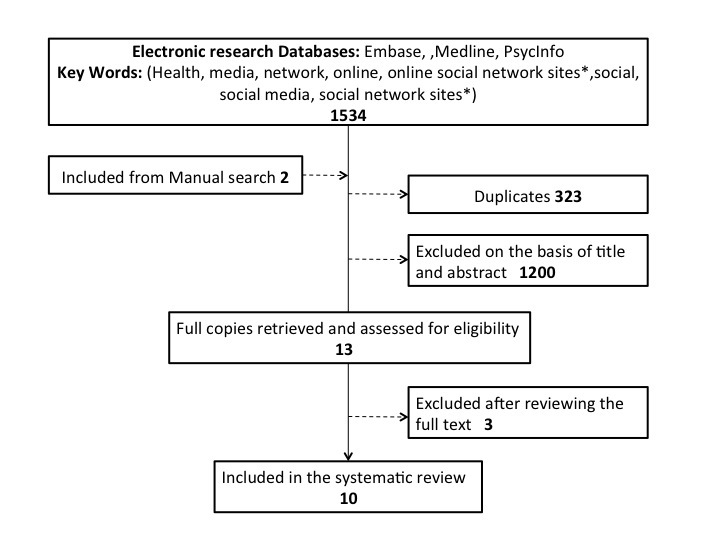
Summary of the systematic literature review process.

A full text assessment was undertaken on the 13 papers that appeared to meet the inclusion criteria based on title and abstract. Of these, 3 were excluded: 2 not employing SNS directly to collect data and 1 discussion paper with no results. A total of 10 papers were reviewed and assessed. Table 1 depicts the search results from each database, and Figure 1 illustrates the review process.

### Included Studies

All included studies were cross-sectional and primarily used self-reported data (Multimedia Appendix 2). While the majority of studies undertook quantitative analysis, 1 study was based on qualitative focus group data. All included studies are defined as high quality after they were assessed by the CDR checklist (Multimedia Appendix 3).

**Table 1:** Database search results.

Database	Database provider	Years searched	Number of citations retrieved (n=1536)
MEDLINE	OVID	1996 to March week 1, 2013	403
PsycInfo	OVID	1996 to March week 2, 2013	142
Embase	OVID	1996 to March week 3 2013	609
MEDLINE	Pubmed	1996 to March week 3, 2013	380
Manual search	–		2

###  Does Health Research Use Social Network Sites as Mode of Collecting Data?

#### Overview

The large number of search results indicate that health research is using SNS in many forms, with the majority of studies investigating the effect of SNS use on health seeking behavior and knowledge. However, there are a limited number of studies that focus on SNS as a tool for research recruitment or data collection; only 10 studies of 1213 looked at this potential.

#### Type of Collected Data

Of the included studies, 9 collected survey data through SNS, while Levine (2011) conducted an online focus group within a SNS [11]. Fenner (2012) used SNS for recruitment, rather than only for data collection [12]. In summary, most of the included studies collected quantitative data, and only one, Levine (2011) collected qualitative data through MySpace and explored possibilities to increase response rates [11]. It is more convenient to collect quantitative data rather than qualitative through SNS, as the latter may require more resources.

#### Data Collection

As this review is looking at SNS as mode to collect health research data, all included studies reached their participants through SNS; however, different approaches were undertaken. Heather (2009) distributed an invitation for an online survey on 8 SNS related to pregnancy and baby health. This approach resulted in 288 valid surveys in a 2-month period [13].

Levine (2011) conducted a focus group by forming a MySpace profile embedded within a chat room and sending invitations to members to join their network; 738 people joined the study’s network. Participant selection was based on reviewing SNS profiles, inviting specific participants that met the inclusion criteria [11]. Although this methodology produced an accurate set of qualitative data, it required several staff members to aid with the recruitment process.

Woolley (2012) was interested in monitoring the impact of a specific Facebook health fan page, “Get Up And Do Something (GUADS)”, on participant health seeking actions and behavior. The study used the fan page to collect data by inviting all fans to participate in an online survey [14]. Although the study reported the use of multiple recruitment methods, no further explanation was given as to the recruitment process.

Of the included studies, 4 used Facebook ads, an online advertising affiliate program, which is a powerful targeted advertisement method. Fenner (2012) assessed the feasibility of recruiting young females using this method. In total, 278 participants were recruited, 139 chose to participate in the online survey, and the remaining 139 opted to physically attend the research center [12]. Ramo (2012), Lohse (2013), and Lord (2011) also used Facebook ads to advertise an online survey to their target populations [15-17].

In addition to Facebook ads, 2 other applications within Facebook were used by health researchers: Facebook Event (a new feature on Facebook where the plugin gives the fan page administrator the ability to add details about upcoming events, eg, Event Name, Location, and Date) and Facebook Poll (a page widget that gives the fan page administrator the ability to add “poll questions” with a voting system and closing date) [18,19]. Finally, Shindel (2012) investigated the association of high risk for sexual dysfunction of women who have sex with women (WSW). Individuals were invited to participate by emailing the entire member list of online social networks catering to WSW [18].

### Is Data Quality Affected by the Mode of Data Collection?

#### Overview

In order to address this question, we looked at data quality indicators such as response rate, completion time, dropout rate, timeliness, missing data, and cost. A comparison was undertaken where appropriate, as studies differed substantially in methodology and population. However, none of the included studies looked at the quality of data in depth or performed any type of analysis regarding quality. Response rate, cost, timeliness, and missing data were reported in some studies.

#### Response Rate

Response rate is defined as the “Number of participants who completed a questionnaire” divided by the ‘Total number of participants who were asked to participate” [20]. The highest response rate 27% (N=2583) was reported by Lord (2011). The survey had been advertised on Facebook for 2 weeks and targeted a young population with no strict inclusion criteria [17].

The lowest response rate, 2.2% (N=738) reported by Levine (2011), resulted from the recruitment method that was used. Participants were invited to a synchronous online focus group (during a specified time). Later changing to asynchronous (ie, no specified time) caused a slight increase to 7.2% (N=250) [11].

The collection of qualitative data is challenging because it requires more effort and staff time as reported by the study authors; in some cases, calculating the response rate was not possible [14]. To summarize (Table 2), the reported response rates ranged between 2% to 27%, with an average of 12%.

**Table 2:** Reported response rates of included studies.

Reference	Study ID	Response rate, %	Participated/Reached
[17]	Lord, 2011	26.67	689/2583
[19]	Cucchetti, 2012	22.80	2414/10584
[16]	Lohse, 2013	17.42	18/465
[14]	Woolley, 2012	11.19	90/804
[15]	Ramo, 2012	10.69	1548/14808
[12]	Fenner, 2012	6.93	551/7940
[11]	Levine, 2011	2.17(synchronous)	16/738
		7.20 (asynchronous)	18/250

#### Cost and Timeliness

Of the studies, 4 used Facebook in their recruitment strategy; 3 were able to report on the cost and timeliness of data collection (Table 3) [12,15,16].

The criteria used by the authors in targeting specific participants will inadvertently affect the number of participant responses. Strasser (2012) set out to recruit 100 participants and closed the survey as soon as this target was met [21]. Evidently, time was not a key factor and the recruitment process would have continued as required.

However, cost is an implication that must be considered. Studies may have a limited budget and could recruit only as many participants as possible within a specific timeframe. Ramo (2012) reported the lowest cost per participant: $4.28 over a 13-month period yielding 1548 participants who were young smokers [15]. Lohse (2013) and Fenner (2012) targeted females within specific age ranges, which could account for the higher costs reported [12,16].

Nevertheless the highest reported cost $20.14 was considered favorable over the cost of traditional methods of recruiting as reported by Fenner (2012) [12].

**Table 3:** Reported cost and timeliness of included studies.

Reference	Study ID	Duration (days)	Participants	Response per day	Cost ($US)		
					Per participant	Per click	Total
[15]	Ramo, 2012	390	1548	4	4.28	0.45	6628.24
[16]	Lohse, 2013	19	62	3	9.26	1.28	596.71
[12]	Fenner, 2012	150	278	2	20.14	0.67	5598.92
[21]	Strasser, 2012	16	100	1.6	–	–	–

#### Other Quality Indicators

Fenner (2012) was the only author to report on missing data. For the demographic questions, this did not exceed 5%, and for remaining questions, this value was less than 8%. The author considered this a positive indicator on the quality of data [12].

Lohse (2013) reported that the completion rate of the survey was 93.5%, which is indicative of good data quality [16]. The validity of the data was reported by Lord (2011) as 76%; however, no further explanation was provided as to how this was assessed [16].

### What Types of Participants Were Reached by Social Networking Sites?

Although SNS is a tool that can be widely used to recruit participants, it may be more effective for certain groups; for example, if targeting an aging population, one has to take into account that this group may not be as computer literate and therefore less likely to use SNS. The types of participant more suited to SNS (Table 4) would be a younger population and those that are “hard to reach”, for example, a homosexual population.

**Table 4:** Types of participants targeted by SNS.

Reference	Study ID	Participant type	Target age, years
[13]	Heather, 2009	Pregnant women	>18
[12]	Fenner, 2012	Female	16-25
[16]	Lohse, 2013	Female	18-45
[18]	Shindel, 2012	WSW	>18
[21]	Strasser, 2012	MSM	No targeted age
[11]	Levine, 2011	Youth	16-24
[17]	Lord, 2011	Youth	18-25
[15]	Ramo, 2012	Young smokers	18-25
[14]	Woolley, 2012	Community	>18
[19]	Cucchetti, 2012	Community	No targeted age

### What Are the Strengths and Limitations of Social Networking Sites?

One of the most reported strengths of SNS is that it is an effective recruitment method. This was stated in 4 studies, which successfully reached young age groups [15], females [12], low-income females [16], and MSM [21]. All these populations were defined by researchers as hard-to-reach groups. Facebook in particular was considered by Ramo (2012) as a successful mechanism to reach and recruit a young age group in smoking-related health research, which is normally a challenge [15]. In addition, Levine (2011), Fenner (2012), and Ramo (2012) reported that SNS proved to be much more cost effective over other traditional methods of recruiting in health research [11,12,15]. SNS can also provide representative and valid data. Fenner (2012) indicated that the SNS sample yielded demographically representative data, and Lord (2011) stated SNS provided a rich pool of qualitative and quantitative valid data [12,17].

Another strength of SNS is that using online focus groups can be an easy and simple process if conducted asynchronously through SNS, which allows one to capture the exact language of participants to analyze [11]. Finally, an important strength of SNS for health surveys and research is the potential for sharing and invitation within the network, enabling surveys to be diffused rapidly between SNS participants [19].

The predominant limitation of SNS for collecting data was that it may introduce self-selection bias, and when there is a self-selection bias usually there is a sample bias and representative and generalizability issues. Strasser (2012) has also stated that self-reported data may affect the reliability and validity of results [21].

## Discussion

### Principal Findings

This comprehensive review addressed our original research questions and found a gap in the literature for evaluating the effectiveness of SNS as a tool in health research. The findings demonstrate that SNS is considered a research tool that can reach wide audiences and simplify the data collection process for health research, especially quantitative data, along with a wide range distribution of surveys reaching many participants through SNS.

SNS is a powerful tool that can provide a wealth of information about research participants and has the potential to capture good quality data, as some of the included studies have shown. However, SNS self-reported data may introduce self-selection bias, sampling bias, or other generalizability/reliability issues. This aspect was not fully investigated in the included studies of this review, which therefore indicates the need for future research or systematic reviews to focus on these issues.

In this review, Facebook was used in 8 out of 10 of the included studies, which indicates its strong potential as a tool for conducting health research. Many features within Facebook empower the research process, for example, Facebook ads, polls, events, and insights. The potential of Facebook needs to be highlighted especially in health research where validity is of utmost importance for research results. Hence, further studies assessing its potential in health research are needed.

### Strengths and Limitations

A number of strengths were highlighted in this review. First, it was an overview of the existence of SNS use in health research literature illustrating the strengths and limitations of this method in data collection. Second, it was a comprehensive and explicit review with broad inclusion criteria that led to a review of 1213 studies, highlighting the gap in the literature regarding the use of SNS as a tool and its effect on data quality.

A limitation of this review is the heterogeneity of the included studies. Although all used SNS to collect data, their individual objectives, populations, and outcomes are unique. Analyses were found to be primarily descriptive.

### Systematic Review Outcomes

SNS can be suitable for health research and was claimed to be an effective tool to collect data, but more research is required to look more closely at its effectiveness as a tool. Comparative research that compares SNS with other data collection modes would be valuable in highlighting differences between the quality of data obtained, costs incurred, and samples obtained. This review indicates that the quality of collected data was not assessed thoroughly; although for surveys and online questionnaires, it led to an acceptable level of validity. Yet, SNS use for data collection proved to be more successful when young age groups were targeted. Finally, Facebook SNS was used in a number of included studies in this review and highlighted that it is a powerful tool that provides multiple features that can be used to improve online health research.

### Conclusions

This review concludes that SNS has a niche in health research, but we need to ascertain how to use it effectively without affecting the quality of research. The field of SNS is growing rapidly and researchers need to take advantage of the strengths of this tool and to avoid its limitations by employing effective research design.

## Multimedia Appendix 1

PRISMA Checklist.

## Multimedia Appendix 2

Summary of included studies.

## Multimedia Appendix 3

Quality assessment of included studies.

## Abbreviations

MSM: men who have sex with men
SNS: social network sites
WSW: women who have sex with women
