# Supplementary material for: Social Network Sites as a Mode to Collect Health Data: A Systematic Review
Source: J Med Internet Res. 2014 Jul 14;16(7):e171. doi: 10.2196/jmir.3050 (PMC4397388; doi:10.2196/jmir.3050)
Supplement: Supplementary file 2 [file jmir_v16i7e171_app2.pdf]

Table 2. Summary of included studies

Reference	Study ID	Title	Objective of study	Target population	Data type	Data collection tool	SNS	Related outcomes
[11]	Heather, 2009	Social media and health: Social support and social capital on pregnancy-related social networking sites.	Whether participation on online social network related to health was associated with health outcomes.	Pregnant women >18	Quantitative	Survey	8 SNS-pregnant health related	• Strategy<br>•Data quality<br>•Limitations
[12]	Levine, 2011	Formative research on MySpace: online methods to engage hard-to-reach populations.	Describe the process of conducting formative research on a popular social networking site, MySpace, in an effort to involve youth of color in design of programmatic content and formats for an Internet intervention.	Youth (16-24)	Qualitative	Focus group	My Space	•Strategy<br>•Data quality<br>•Strength
[13]	Woolley, 2012	Efficacy of a health-related Facebook social network site on health-seeking behaviors.	Determine the impact of a health-related Facebook fan page on health-seeking actions, thoughts, and behaviors.	>18	Quantitative	Survey	Facebook	•Strategy<br>•Data quality<br>•Limitation
[14]	Fenner, 2012	Web-based recruiting for health research using a social networking site: An exploratory study.	To assess the feasibility of recruiting young females using targeted advertising on the social networking site Facebook.	Females (16- 25)	Quantitative	Survey	Facebook	•Strategy<br>•Data quality<br>•Strength
[15]	Ramo, 2012	Broad reach and targeted recruitment using Facebook for an online survey of young adult substance use.	Examined Facebook as a mechanism to reach and survey young adults about tobacco and other substance use.	Young smokers (18-25)	Quantitative	Survey	Facebook	•Strategy<br>•Data quality<br>•Strength<br>•Limitation

[16]	Shindel, 2012	An Internet survey of demographic and health factors associated with risk of sexual dysfunction in women who have sex with women (WSW).	To investigate the associations of high risk for sexual dysfunction in an Internet cohort of WSW.	WSW >18	Quantitative	Survey	SNS for WSW + Facebook	•Strength<br>•Limitation
[17]	Strasser, 2012	Feasibility study of social media to reduce intimate partner violence among men who have sex with men (MSM) in metro Atlanta, Georgia.	Examine the feasibility of enrolling 100 gay men from Atlanta into an Intimate Partner Violence survey study. The survey was administered via Facebook.	MSM	Quantitative	Survey	Facebook	•Strategy<br>•Data quality<br>•Strength<br>•Limitation
[18]	Lohse, 2013	Facebook is an effective strategy to recruit low-income women to online nutrition education.	Examine Facebook as a strategy to recruit low-income women, within a specific age and geographic location, to participate in an online nutrition education program.	Females (18-45)	Quantitative	Survey	Facebook	•Strategy<br>•Data quality<br>•Strength
[19]	Cucchetti, 2012	The use of social networking to explore knowledge and attitudes toward organ donation in Italy.	Investigate issues regarding organ donation.	Community, no targeted age.	Quantitative	Survey	Facebook	•Strategy<br>•Data quality<br>•Strength<br>•Limitation
[20]	Lord, 2011	Connecting to young adults: an online social network survey of beliefs and attitudes associated with prescription opioid misuse among college students.	(1) Assess the feasibility of implementing a survey study about prescription medication misuse with college students on Facebook<br>(2) Identify the characteristics, motivations, beliefs and attitudes associated with experimental versus regular misuse of prescription opioid analgesic medications.	young adults (18-25)	Quantitative	Survey	Facebook	•Strategy<br>•Data quality<br>•Strength<br>•Limitation
