# Supplementary material for: Social Network Sites as a Mode to Collect Health Data: A Systematic Review
Source: J Med Internet Res. 2014 Jul 14;16(7):e171. doi: 10.2196/jmir.3050 (PMC4397388; doi:10.2196/jmir.3050)
Supplement: Supplementary file 3 [file jmir_v16i7e171_app3.pdf]

Table 3. Quality assessment of included studies.

Reference	Study ID	Indicator								Quality indicators met
		1.Appropriate research design?	2.Appropriate recruitment strategy?	3.Response rate?	4.Sample representative?	5.Objective and reliable measures?	6.Power calculation or justification of numbers?	7.Appropriate statistical analysis?	8.Evidence of bias?	
[11]	Heather, 2009	1	1	-	1	1	-	1	-	5/8
[12]	Levine, 2011	1	1	1	-	1	-	1	1	6/8
[13]	Woolley, 2012	1	1	1	-	1	1	1	-	6/8
[14]	Fenner, 2012	1	1	1	1	1	1	1	-	6/8
[15]	Ramo, 2012	1	1	1	1	1	1	1	-	7/8
[16]	Shindel, 2012	1	1	-	1	1	1	1	1	6/8
[17]	Strasser, 2012	1	1	-	-	1	-	1	-	5/8
[18]	Lohse, 2013	1	1	1	1	1	1	1	-	7/8
[19]	Cucchetti, 2012	1	1	1	1	1	1	1	-	7/8
[20]	Lord, 2011	1	1	1	1	1	1	1	-	7/8
	Total	10/10	10/10	7/10	7/10	10/10	7/10	10/10	2/10	----
